# Effect of fucoidan on ethanol-induced liver injury and steatosis in mice and the underlying mechanism

**DOI:** 10.29219/fnr.v65.5384

**Published:** 2021-04-20

**Authors:** Meilan Xue, Hui Liang, Zhitong Zhou, Ying Liu, Xinjia He, Zheng Zhang, Ting Sun, Jia Yang, Yimin Qin, Kunpeng Qin

**Affiliations:** 1 Department of Biochemistry and Molecular Biology, Basic Medical College, Qingdao University of Medicine, Qingdao, PR China; 2 The Institute of Human Nutrition, College of Public Health, Qingdao University of Medicine, 308# Ningxia Road, Qingdao, 266071, PR China; 3 Food Science Department, University of Guelph, Guelph, Ontario, Canada; 4 Oncology Department, The Affiliated Hospital of Qingdao University, Qingdao, PR China; 5 State Key Laboratory of Bioactive Seaweed Substances, Qingdao Brightmoon Seaweed Group Co., Ltd., Qingdao, China

**Keywords:** fucoidan, ethanol-induced liver injury, AMPKa1/SIRT1 pathway, gut microbiota–bile acid–liver axis

## Abstract

**Background:**

Alcoholic liver disease is caused as a result of chronic alcohol consumption. In this study, we used an alcoholic liver injury mouse model to investigate the effect of fucoidan on ethanol-induced liver injury and steatosis and the underlying mechanisms.

**Methods:**

All mice were randomly divided into four groups: 1) control group, 2) model group, 3) diammonium glycyrrhizinate treatment group (200 mg/kg body weight), and 4) fucoidan treatment group (300 mg/kg body weight). Administration of ethanol for 8 weeks induced liver injury and steatosis in mice.

**Results:**

Fucoidan treatment decreased serum alanine aminotransferase activity, serum total cholesterol levels, and hepatic triglyceride levels, and improved the morphology of hepatic cells. Fucoidan treatment upregulated the expression of AMPKα1, SIRT1, and PGC-1α and inhibited the expression of ChREBP and HNF-1α. The levels of hepatic IL-6 and IL-18 were significantly decreased in the fucoidan group. Further, the levels of cytochrome P450-2E1 (CYP2E1), glucose-regulated protein (GRP) 78, and 3-nitrotyrosine (3-NT) in hepatic tissues were reduced in the fucoidan group as compared to the model group. Fucoidan significantly reversed the reduction of ileac Farnesoid X receptor (FXR) and fibroblast growth factor 15 (FGF15) levels induced by alcohol-feeding and reduced CYP7A1 (cholesterol 7α-hydroxylase) expression and total bile acid levels in the liver tissue. In addition, fucoidan regulated the structure of gut flora, with increased abundance of *Prevotella* and decreased abundance of *Paraprevotella* and *Romboutsia* as detected by 16S rDNA high-throughput sequencing.

**Conclusion:**

Fucoidan inhibited alcohol-induced steatosis and disorders of bile acid metabolism in mice through the AMPKα1/SIRT1 pathway and the gut microbiota–bile acid–liver axis and protected against alcohol-induced liver injury in vivo.

## Popular scientific summary

- This study was to investigate the effect and its possible mechanism of fucoidan on ethanol-induced liver injury and steatosis in mice.
- The results suggested that fucoidan inhibited alcohol-induced steatosis, inflammation and oxidative stress through the AMPK/SIRT1 signaling pathway, and improved the disorder of bile acid metabolism by regulating gut microbiota-bile acid-liver axis.

Alcohol abuse has become a serious public health concern, leading to approximately 3.3 million deaths every year. A report by the World Health Organization (WHO) revealed that alcohol abuse accounts for 5.9% of all deaths (1). Chronic alcohol consumption results in alcoholic liver disease (ALD) (2). In addition, long-term alcohol exposure leads to hepatic steatosis and inflammation, which aggravates liver injury (3)

The mechanisms of ALD pathogenesis are complex, and the occurrence of lipid metabolism disorder, oxidative stress, and inflammatory reaction have been confirmed (4, 5). Alcohol-inducible cytochrome P450-2E1 (CYP2E) can oxidize excessive amounts of ethanol to acetaldehyde and produce significant amounts of reactive oxygen species (ROS), which leads to mitochondrial damage and acceleration of fatty liver formation (6, 7). ROS also leads to activation of Kupffer cells (KCs), which constitute 80–90% of the tissue macrophages. KCs play an important role in inflammation and hepatic homeostasis. Activated KCs are important in the pathogenesis and the progression of ALD as they induce an inflammatory cascade and release interleukin-1β (IL-1β), IL-6, IL-18, and other pro-inflammatory cytokines to aggravate liver damage (8, 9). Chronic alcohol consumption may have an additional impact on the endoplasmic reticulum and mitochondria (10). Alcohol directly affects the normal functioning of these organelles and even causes damage to these organelles. The levels of 3-nitrotyrosine (3-NT) and glucose-regulated protein (GRP) 78 were increased as a result of chronic alcohol consumption, suggesting that 3-NT and GRP78 were closely associated with ALD (11, 12). Among the different complex stages, development of liver steatosis is the initial stage of ALD and is a reversible condition. Long-term ethanol exposure can result in irreversible conditions such as steatohepatitis, fibrosis, cirrhosis, and even hepatocellular carcinoma.

It has been reported that fucoidan extract protects against non-alcoholic fatty-liver damage (NAFLD) because of the activation of the AMPK/SIRT1/PGC1α (peroxisome proliferator-activated receptor-γ coactivator-1α) pathway in mice, which prevents lipotoxicity-related oxidative stress and inflammation (13). Therefore, fucoidan has a potential therapeutic effect on liver injury. Fucoidan is extracted from brown seaweeds and belongs to the class of L-fucose enriched sulfated polysaccharides. It has been reported that fucoidan exhibits anti-aggregative, anti-inflammatory, and antioxidant properties (14). Moreover, fucoidan could improve antioxidant status and ameliorate steatohepatitis (15, 16). Meenakshi et al. (17) found that treatment with fucoidan suppressed oxidative stress in rats exposed to alcohol. Fucoidan increased the levels of enzymatic antioxidants and decreased the levels of lipid peroxidation markers. Kim et al. (18) reported that fucoidan treatment inhibited inflammatory response in the liver and protected against ALD in mice.

However, the molecular mechanism by which fucoidan protected alcoholic liver damage is not well understood. The effect of fucoidan on the lipid metabolism disorder, lipotoxicity-related oxidative stress, and the gut flora–bile acid–liver axis in ALD and the underlying mechanisms remain unclear. Therefore, in this study, we generated an alcoholic liver injury mouse model and administered fucoidan obtained from the brown seaweed *Fucus vesiculosus* to the mice to study the protective effect of fucoidan against ALD in vivo. The mechanism underlying the effect of fucoidan on the gut flora–bile acid–liver axis was also determined.

## Materials and methods

### Animals

The present work was approved by the Review Committee for the Use of Human or Animal Subjects of the Medical College of Qingdao and was conducted in accordance with the Helsinki Declaration of 1975, as revised in 2008.

Specified-pathogen-free (SPF) male C57BL/6J mice (8 weeks old) were provided by Weitong Lihua Experimental Animal Technology Co., Ltd. (Beijing, China).

### Fucoidan preparation

Fucoidan was extracted from *Fucus vesiculosus* as described in our previous study (19). The fucoidan sample predominantly contained fucose (42.5%) and mannose (6.3%), and the content of sulfates was 32.1%. The purity of the fucoidan sample was about 92%.

### Experimental design

After an acclimatization period of 1 week with free access to food and water, the mice were randomly divided into four groups: 1) control group, 2) model group, 3) diammonium glycyrrhizinate (DG) treatment group (DG group), and 4) fucoidan treatment group (fucoidan group). Each group contained eight mice. The animals in the model group, DG group, and fucoidan group were administered 50% (v/v) ethanol by gavage at a concentration of 8 mL/kg body weight every day for 2 weeks and 12 mL/kg body weight for the next 6 weeks. Meanwhile, the mice in the control group received equal volumes of 0.9% normal saline (NS) instead of ethanol. After 2 h of ethanol administration, DG suspensions prepared in NS were administered to the DG group animals (200 mg/kg body weight) and fucoidan dissolved in NS was administered to the fucoidan group animals (300 mg/kg body weight) intragastrically. Dose levels of DG and fucoidan were selected according to previous studies (20–23). Meanwhile, control and model group mice were administered equal volumes of NS. DG (>98% purity) was purchased from the Chia Tai Tianqing Pharmaceutical Group Co., Ltd. (Nanjing, Jiangsu, China).

After 8 weeks of treatment, fresh feces were collected and immediately stored at −80 °C for subsequent analysis. Next, all mice were anesthetized by intraperitoneal injection of 40 mg/kg sodium pentobarbital after fasting for 12 h. Blood samples, liver tissues, and ileum tissues were collected and flash frozen in liquid nitrogen.

### Liver histological analysis

One portion of the liver tissues was fixed with 4% paraformaldehyde and, after 24 h, embedded in paraffin. Liver sections (4 μm-thick) were cut, deparaffinized in xylene, rehydrated in gradients of ethanol, and stained with hematoxylin and eosin (HE). The stained sections were observed under a light microscope (Olympus CX23, Tokyo, Japan).

Ultrastructural analysis of the liver was performed using transmission electron microscopy. Liver tissues were cut into 1 × 2 mm sections and fixed in 2.5% glutaraldehyde for 2 h. Next, the tissues were fixed with 1% osmic acid for 1 h and dehydrated in graded acetone. After washing with phosphate buffered saline (PBS, pH 7.2), the tissues were embedded in ethoxyline resin and solidified in a thermostat. Slices of 50–70 nm were cut and double stained with 3% uranyl acetate for 30 min and lead citrate for 15 min. All slices were observed under a transmission electron microscope (JEOL, Tokyo, Japan).

### Analysis of serum lipid and liver lipid levels

The levels of total cholesterol (CHOL), triglyceride (TG), total bile acid (TBA), low-density lipoprotein cholesterol (LDL-CH), and high-density lipoprotein cholesterol (HDL-CH) in serum and hepatic homogenates were quantified using commercial enzyme-linked immunosorbent assay (ELISA) kits according to the manufacturer’s instructions (Nanjing Jiancheng Co., Nanjing, China).

### Measurement of serum aminotransferase and hepatic inflammatory cytokine levels

The levels of aspartate aminotransferase (AST) and alanine aminotransferase (ALT) in serum were determined using ELISA kits. Hepatic IL-1β, IL-6, and IL-18 levels were also estimated using ELISA kits. All ELISA kits were purchased from Nanjing Jiancheng Co. (Nanjing, China).

### Western blotting

RIPA buffer containing polymethyl-sulfonyl fluoride (PMSF) was used for homogenization and lysis of liver and ileum tissues. The total protein content in each sample was detected using a Pierce™ Rapid Gold BCA (bicinchoninic acid) Protein Assay Kit (Thermo Scientific, Shanghai, China). Samples containing 30 µg of proteins were resolved using 12% sodium dodecyl sulfate–polyacrylamide gel electrophoresis (SDS-PAGE). The protein bands were electro-transferred onto polyvinylidene fluoride (PVDF) membranes (Solarbio Science & Technology, Beijing, China). Next, the membranes were incubated with 5% non-fat dry milk for 1 h to block all nonspecific sites, followed by incubation with primary antibodies at 4°C overnight.

Anti-Grp78 (66574-1-Ig, 1:500), anti- HNF-1α (anti-hepatocyte nuclear factor-1α; 22426-1-AP, 1:1,000), and anti-CYP2E1 (19937-1-AP, 1:1,000) antibodies were obtained from Proteintech (Rosemont, IL, USA). Anti-3-NT (ab61392, 1:800), anti-ChREBP (anti-carbohydrate response element binding protein; ab92809, 1:800), and anti-FGF15 (anti-fibroblast growth factor 15; ab229630, 1:500) antibodies were obtained from Abcam (Cambridge, UK). Anti-SIRT1 (A0230, 1:800), anti-p-AMPKα1 (AP0116, 1:1,000), anti-PGC-1α (A11971, 1:1,000), and anti-FXR (anti-Farnesoid X receptor; A8320, 1:1,000) antibodies were purchased from ABclonal Biotechnology (Harrogate, UK). Anti-CYP7A1 (anti- cholesterol 7α-hydroxylase; sc-518007, 1:1,500) and anti-β-actin (sc08432, 1:1,500) antibodies were purchased from Santa Cruz Biotechnology (CA, USA).

After incubation with primary antibodies, the membranes were washed twice with tris-buffered saline (TBS) and incubated with 1:1,000 or 1:800 diluted horseradish peroxidase conjugated secondary antibodies for 1 h. Finally, an Electro-Chemi-Luminescence (ECL) Western blotting kit (BioVision, San Francisco, USA) was used to carry out detection. The relative densities of the protein bands were evaluated using the image analysis system (Bio-Rad Gel Doc 2000, USA).

### Immunofluorescence analysis

Frozen liver tissues were fixed in 10% formaldehyde and embedded in paraffin. Tissue slices of 5 μm were cut for analysis, and the slices were blocked with 3% bovine serum albumin (BSA) and incubated with primary anti-CYP2E1 antibody overnight at 4°C. Next, the slices were washed thrice with TBS followed by incubation with goat anti-mouse IgG H&L (Alexa Fluor® 488) (ab150113, 1: 60) at 37°C in the dark for 30 min. Finally, the sections were incubated with 4’,6-diamidino-2-phenylindole (DAPI) for 15 min and observed under Leica microscope (Leica TCS SP8, Solms, Germany).

### Analysis of fecal bile acids

Fecal bile acid analysis was performed using liquid chromatography–mass spectrometry (LC-MS) as previously described (24). Briefly, the fecal samples were dried, lyophilized, and reconstituted with 75% ethanol. After homogenization, the samples were centrifuged at 10,000 rpm for 10 min at 4°C. The supernatants were evaporated under vacuum and the residue was reconstituted in the assay mobile phase for bile acid determination. The injection volume was 1 µL. The temperature of the auto sampler and the column was 12 and 65°C, respectively.

### 16S rDNA gene sequencing

The microbial community structures of 15 fecal samples (five samples were randomly selected from each group) were analyzed at the Realbio Genomics Institute (Shanghai, China). The Illumina HiSeq platform was used to perform 16S rDNA gene sequencing as previously described (23). Briefly, total genomic DNA was extracted using a QIAamp DNA Stool Mini Kit (Qiagen, Hilden, Germany). The primers F341 (5′-CCTACGGGRSGCAGCAG-3′) and R806 (5′-GGACTACVVGGGTATCTAATC-3′) were used to amplify the V3–V4 region. After quality control and clustering analysis at 97% similarity level, operational taxonomic units (OTUs) were generated from the raw data using Uparse software package (Uparse v7.0.1001).

### Statistical analyses

The results are expressed as mean ± standard deviation (SD). Statistical analyses were performed using GraphPad Prism 7.0 (GraphPad Software Inc., La Jolla, CA, USA). One-way analysis of variance (ANOVA) was used to compare the means of multiple groups. Dunnet’s posthoc test was used to determine the significance of differences between two groups. R3.1.0 Differences was used to perform principal component analysis (PCA) and heat map analysis. The differences were considered statistically significant at a *P*-value less than 0.05.

## Results

### Effect of fucoidan on weight gain, serum aminotransferase, and hepatic inflammatory factors

**Fig. 1:**
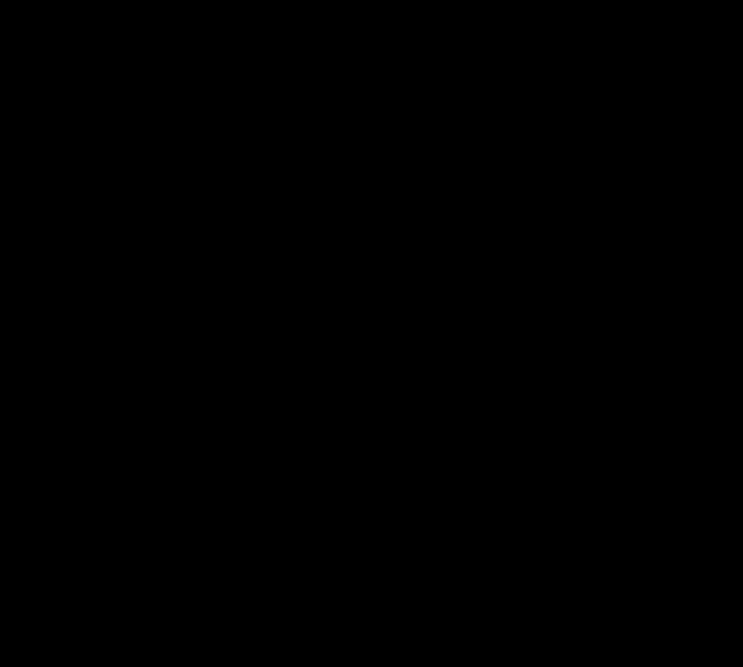
Weight gain and levels of serum aminotransferase and hepatic inflammatory factors. (a) Weight gain curve. There were no significant differences in the initial body weight of the mice. Over a period of 8 weeks, only control group mice exhibited weight gain. At the end of the experiment, ethanol-fed (model group) mice showed a significant decrease in the final body weight as compared to control mice. (b, c) Serum ALT (b) and AST (c) levels. Model group mice exhibited significantly higher serum AST and ALT levels as compared to control group mice. Diammonium glycyrrhizinate treatment decreased the levels of serum AST and ALT, and fucoidan treatment decreased the levels of serum ALT as compared to the model group (*P* < 0.05). (d–f) Levels of hepatic IL-1β (d), IL-6 (e), and IL-18 (f). Hepatic IL-1β, IL-6, and IL-18 levels were higher in the model group than in the control group. Compared to the model group, treatment with diammonium glycyrrhizinate decreased the levels of hepatic IL-1β and IL-6, and treatment with fucoidan significantly decreased the levels of hepatic IL-6 and IL-18. Note: Data are represented as mean ± SD. *n* = 8 in each group. *P ˂ 0.05 versus control; **P ˂ 0.01 versus control; ^#^ *P* ˂ 0.05 versus model.

As shown in Fig. 1a, there were no significant differences in body weight of the mice at the beginning of the experiment. Over a period of 8 weeks, mice in the control group gained weight. By the end of the study, there was a significant decrease in the final body weight of model group mice as compared to control group mice (*P* < 0.05). Compared to the model group, the final body weight of the DG group and fucoidan group mice was slightly higher, however, the difference was not statistically significant (*P* > 0.05).

In addition, we detected serum ALT and AST levels, which are major markers of liver damage. Ethanol feeding significantly increased the levels of ALT and AST in serum (3.2-fold and 1.8-fold, respectively, *P* < 0.05) as compared to the control group. DG group mice exhibited lower serum ALT and AST levels as compared to model group mice (*P* < 0.05). Similarly, fucoidan treatment decreased serum ALT levels by 46.8% in the fucoidan group as compared to model group mice (*P* < 0.05, Fig. 1b and c). These results indicated that fucoidan treatment alleviated ethanol-induced liver injury and the effect was similar to that of DG.

As shown in Fig. 1d–f, ethanol feeding increased hepatic IL-6, IL-1β, and IL-18 levels as compared to the control group (*P* < 0.05). Compared to the model group, DG treatment decreased hepatic IL-1β and IL-6 levels, while fucoidan treatment significantly decreased hepatic IL-6 and IL-18 levels (*P* < 0.05, Fig. 1d–f).

### Effect of fucoidan on liver histopathology

**Fig. 2:**
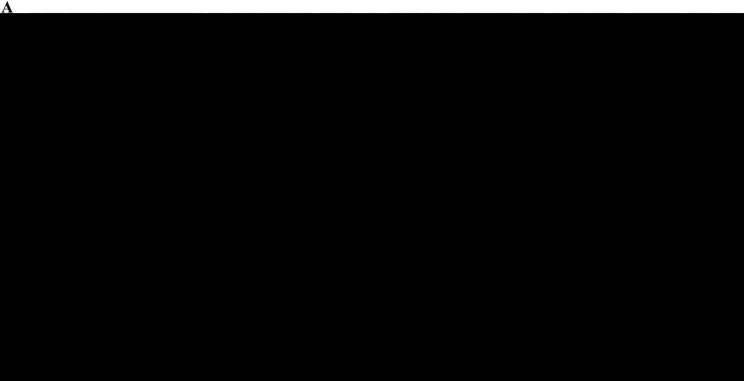
Pathological changes in the liver. (a) Histopathological analysis of the liver. Model group mice showed an irregular arrangement of hepatocytes, extensive fat droplets, Mallory bodies, and inflammatory infiltration in the liver tissues. Mice in the DG group and fucoidan group showed reduced lipid accumulation and less inflammatory infiltration in the liver as compared to model group mice. (b) Ultrastructural analysis of the liver using transmission electron microscopy. Model group hepatocytes exhibited irregular shape, increased lipid droplets, swollen and deformed mitochondria with fuzzy mitochondrial cristae, and degranulated endoplasmic reticulum. In the DG group and fucoidan group, the hepatic cell morphology was improved, with neatly arranged endoplasmic reticulum and clear mitochondrial cristae.

Histopathological observation of the liver revealed hepatic cords radiating around the central vein in each lobule, clear hepatic lobule structures, and no obvious steatosis in the control group. However, ethanol feeding resulted in irregular arrangements of hepatic cords, Mallory bodies, inflammatory infiltration, and extensive accumulation of lipid droplets in liver tissues, which were not observed in the liver tissues of control group animals (Fig. 2a). However, DG group and fucoidan group mice showed less lipid accumulation and reduced inflammatory infiltration as compared to model group mice.

As shown in Fig. 2b, ultrastructural analysis of the liver revealed normal hepatic cells with abundant organelles, less lipid droplets, large numbers of well-formed mitochondria, and neatly arranged rough endoplasmic reticulum in control group mice. Model group hepatocytes exhibited an irregular shape, large numbers of lipid droplets, swollen and deformed mitochondria with fuzzy mitochondrial cristae, and degranulated endoplasmic reticulum. In the DG and fucoidan groups, the morphology of hepatic cells was improved, with neatly arranged endoplasmic reticulum and clear mitochondrial cristae.

### Effects of fucoidan on serum lipid and hepatic lipid profiles

**Fig. 3:**
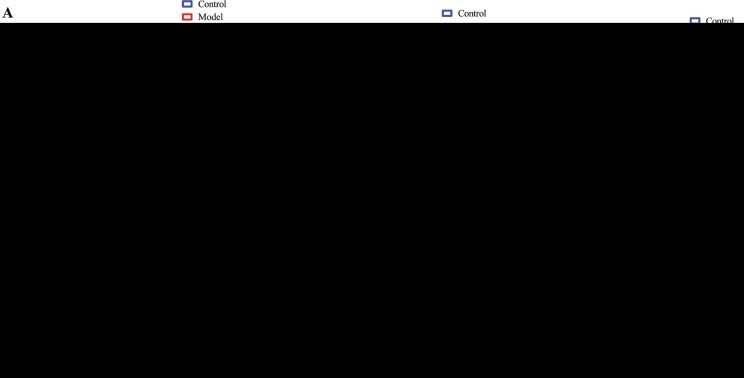
Serum and hepatic lipid profiles. (a) Serum lipid profiles. (b) Hepatic lipid profiles. Compared to the control group, ethanol feeding significantly increased the levels of serum TG, CHOL, LDL-CH, and TBA. The levels of hepatic TG, LDL-CH, and TBA in the model group were also higher than that in the control group. However, fucoidan treatment decreased the levels of serum CHOL and hepatic TG and TBA and increased hepatic HDL-CH levels as compared to the model group. Note: Data are represented as mean ± SD. *n* = 8 in each group. *P ˂ 0.05 versus control; **P ˂ 0.01 versus control; ^#^*P* ˂ 0.05 versus model.

Model group mice that were fed ethanol showed a significantly increased serum TG, CHOL, LDL-CH, and TBA levels as compared to control group mice (*P* < 0.05, Fig. 3 and S1 Excel). Similarly, hepatic TG, LDL-CH, and TBA levels were also increased in model group mice as compared to control group mice (*P* < 0.05 or *P* < 0.01). However, fucoidan treatment decreased serum CHOL levels and hepatic TG and TBA levels and increased hepatic HDL-CH levels as compared to the model group (*P* < 0.05).

### Effects of fucoidan on the protein expression levels of CYP2E1, Grp78, and 3-NT in mice liver

**Fig. 4:**
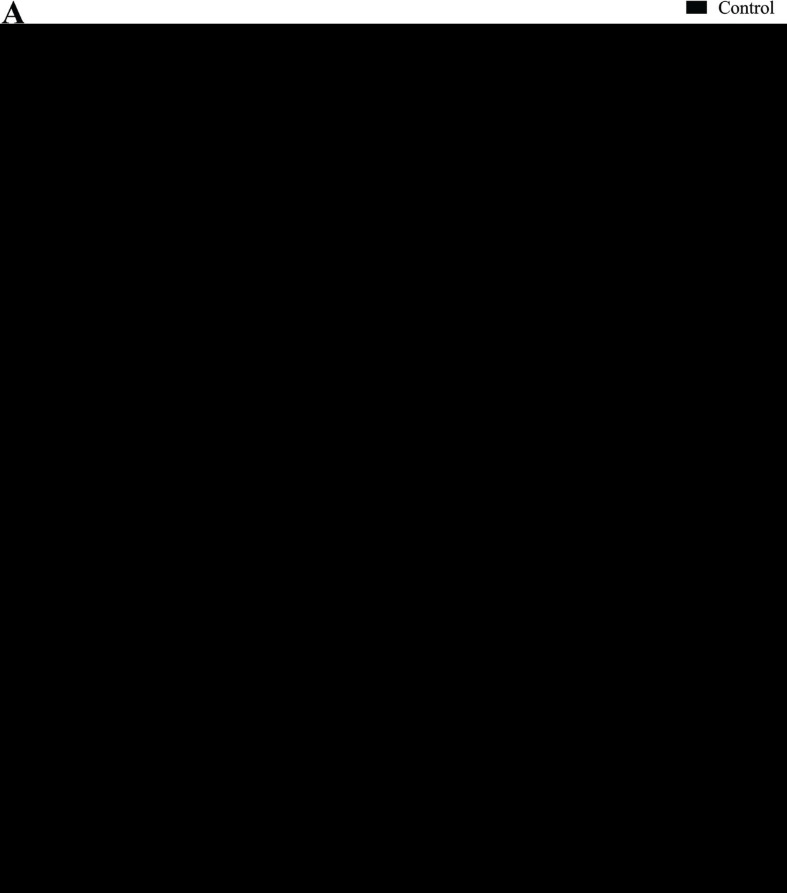
Protein expression levels of CYP2E1, Grp78, and 3-NT in the liver. (a) Western blot analysis of the expression of CYP2E1, Grp78, and 3-NT in liver tissues. Model group mice showed significantly higher levels of CYP2E1, Grp78, and 3-NT compared to control group mice. Compared to the model group, the levels of hepatic CYP2E1, Grp78, and 3-NT were markedly downregulated in the fucoidan group. Note: *P ˂ 0.05 versus control; **P ˂ 0.01 versus control; ^#^*P* ˂ 0.05 versus model. (b) Immunofluorescence analysis of CYP2E1. Liver tissues of fucoidan group mice displayed significantly weaker CYP2E1 staining compared to that of model group mice.

We detected the expression levels of oxidative stress markers in liver tissues using western blot analysis. As shown in Fig. 4a, model group mice exhibited higher levels of CYP2E1, Grp78, and 3-NT as compared to control group mice (*P* < 0.05 or *P* < 0.01). However, the levels of CYP2E1, Grp78, and 3-NT were markedly downregulated in fucoidan group mice as compared to model group mice (*P* < 0.05).

As shown in Fig. 4b, immunofluorescence analysis revealed high expression of CYP2E1 in ethanol-fed mice as compared to control group mice. However, weaker CYP2E1 staining was observed in liver tissues of mice after fucoidan treatment. These results suggest that fucoidan treatment may suppress ethanol-induced liver injury.

### Effect of fucoidan on the AMPK/SIRT1 signaling pathway

**Fig. 5:**
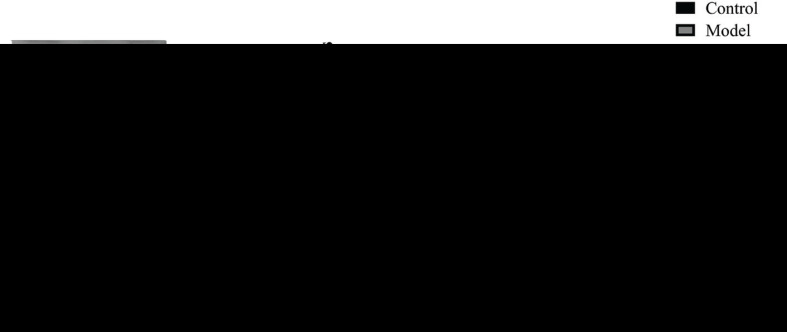
Effect of fucoidan on the AMPK/SIRT1 signaling pathway. Western blot analysis showed that the expression of p-AMPKα, SIRT1, and PGC-1α was suppressed by ethanol feeding. However, treatment with fucoidan significantly reversed this effect of ethanol on hepatic p-AMPKα, SIRT1, and PGC-1α levels. In addition, ethanol-fed mice had significantly higher ChREBP and HNF-1α levels compared to control mice but treatment with fucoidan reduced these levels. Note: *P ˂ 0.05 versus control; **P ˂ 0.01 versus control; ***P ˂ 0.001 versus control; ^#^*P* ˂ 0.05 versus model.

To understand the mechanism underlying the protective effects of fucoidan against ethanol-induced liver injury and steatosis, we analyzed the hepatic expression of p-AMPKα, SIRT1, ChREBP, PGC-1α, and HNF-1α, which regulate hepatic fat metabolism and inflammatory response. Western blot results showed that ethanol feeding suppressed the levels of p-AMPKα, SIRT1, and PGC-1α, thereby promoting alcoholic fatty liver. However, treatment with fucoidan reversed the effect of ethanol on p-AMPKα, SIRT1, and PGC-1α expression (Fig. 5). Similarly, ethanol-fed mice exhibited significantly higher hepatic ChREBP and HNF-1α levels compared to control group mice, but fucoidan group mice showed reduced levels of ChREBP and HNF-1α as compared to model group mice (*P* < 0.05).

### Effect of fucoidan on fecal bile acid levels and the bile acid–FXR–FGF15 axis

**Fig. 6:**
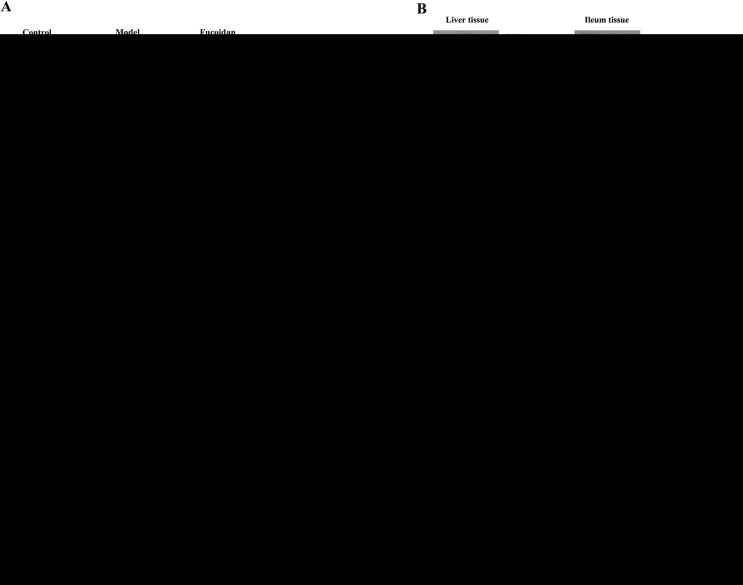
Effect of fucoidan on fecal bile acid profiles and the bile acid–FXR–FGF15 axis. (a) Fecal bile acid analysis using LC-MS. Ethanol-fed mice showed significantly higher fecal CA and MCA levels but lower DCA, TDCA, and GDCA levels as compared to control mice. However, fucoidan treatment reduced CA and MCA levels but increased TDCA levels as compared to the model group. The bile acid profiles of the fucoidan intervention group were similar to those of the control group. (b) Western blot analysis of the expression of ileac FXR and FGF15 and hepatic CYP7A1. Ethanol-fed mice exhibited reduced levels of ileac FXR and FGF15 and increased expression of hepatic CYP7A1. However, treatment with fucoidan significantly reversed this effect of ethanol on ileac FXR and FGF15 and hepatic CYP7A1 levels. Note: *P ˂ 0.05 versus control; **P ˂ 0.01 versus control; ^#^*P* ˂ 0.05 versus model.

To assess the effect of fucoidan on bile acid metabolism, fecal bile acid levels were detected using HPLC. As shown in Fig. 6a and S2 Excel, alcohol-fed mice showed significantly higher fecal cholic acid (CA) and muricholic acid (MCA) levels but lower deoxycholic acid (DCA), taurodeoxycholic acid (TDCA), and glycodesoxycholic acid (GDCA) levels compared to control group mice. Fucoidan treatment reduced CA and MCA levels but increased TDCA levels as compared to the model group. The bile acid profiles of the fucoidan intervention group were similar to those of the control group. These data indicate that fucoidan could alleviate ethanol-induced disorders of bile acid metabolism.

It has been reported that conjugated bile acids induce the activity of FXR, and FGF15 regulates CYP7A1 (the rate-limiting enzyme in bile acid synthesis) level via a negative feedback mechanism in the liver (25). Therefore, we evaluated the protein expression levels of ileac FXR and FGF15 and hepatic CYP7A1 by western blot analysis. As shown in Fig. 6b, ethanol feeding reduced the levels of FXR and FGF15 in ileum tissues and increased the expression of CYP7A1 in liver tissues as compared to the control group. However, fucoidan treatment reversed the effect of alcohol on ileac FXR and FGF15 levels and hepatic CYP7A1 levels (*P* < 0.05).

### Effect of fucoidan on the gut flora of mice

**Fig. 7:**
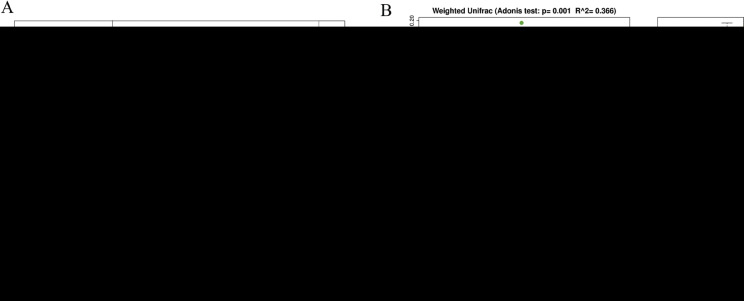
Diversity analysis of the gut flora of mice.(a) PCA analysis; (b) Weighted Unifrac PCOA analysis (Adonis test). The diversity analysis based on OTU abundance showed that there was a significant difference in the structure of gut flora among the three groups. C: control group; M: model group; F: fucoidan group.

The V3–V4 region of 16S rDNA in mice was analyzed by using Illumina Miseq high-throughput sequencing technology. PCA and weighted Unifrac principal coordinate analysis (PCOA) analysis (Adonis test) based on OTU abundance were performed. The diversity analysis showed that there was a significant difference in the structure of gut flora among the control, model, and fucoidan treatment groups (*R* = 0.366, *P* = 0.001, Fig. 7).

**Fig. 8:**
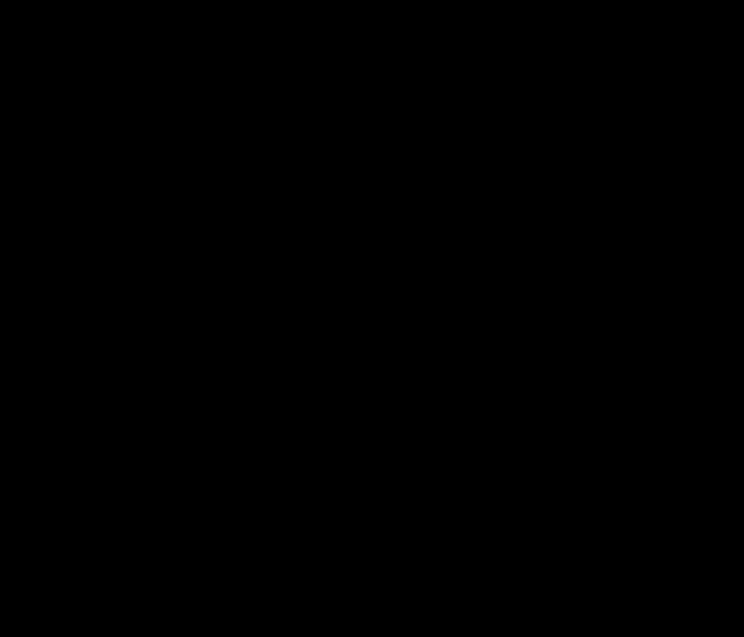
Composition of the gut flora of mice. (a) Analysis of the composition of gut flora at the phylum level. Alcohol feeding increased the abundances of Proteobacteria and Euryarchaeota as compared to the control group. After treatment with fucoidan, the abundance of Firmicutes was lower compared to that in the model group, and the abundance ratio of Firmicutes/Bacteroidetes was also lower compared to that in the control and model groups. (b) Analysis of the composition of gut flora at the genus level. (c) Heat map analysis of the composition of gut flora. Among the top 20 genera, ethanol feeding decreased the abundances of *Prevotella*, *Lactobacillus*, and *Ruminococcus*, and increased the abundances of *Alloprevotella*, *Escherichia*, *Paraprevotella*, *Methanobrevibacter,* and *Romboutsia* as compared to the control group. The genus distribution of fucoidan group mice was similar to that of control group mice. Treatment with fucoidan increased the abundance of *Prevotella*, and decreased the abundances of *Paraprevotella*, *Romboutsia,* and *Clostridium sensu stricto.* C: control group; M: model group; F: fucoidan group.

Bacteroidetes, Firmicutes and Proteobacteria comprised the main phyla. However, the relative abundances of each phylum were different among the three groups (Fig. 8a and S3 Excel). The abundances of Proteobacteria and Euryarchaeota were higher in ethanol-fed mice as compared to control group mice. Fucoidan treatment resulted in reduced abundance of Firmicutes (29.3%), which was significantly lower than that in the model group (40.5%, *P* < 0.05). The abundance of Bacteroidetes in the fucoidan group increased to 61.1% as compared to the model group, but the difference was not statistically significant (*P* > 0.05). However, the abundance ratio of Firmicutes/Bacteroidetes was 0.48 in the fucoidan group, which was lower than that in the model group (0.80) (*P* < 0.05).

As shown in Fig. 8b and c and S4 Excel, the composition of gut flora was also analyzed at the genus level. Among the top 20 genera, ethanol feeding decreased the abundances of *Prevotella*, *Lactobacillus*, and *Ruminococcus*, and increased the abundances of *Alloprevotella*, *Escherichia*, *Paraprevotella*, *Methanobrevibacter, Fusobacterium, Turicibacter,* and *Romboutsia* as compared to the control group (*P* < 0.05 or *P* < 0.01). The genus distribution of fucoidan group mice was similar to that of control group mice. Fucoidan treatment increased the abundance of *Prevotella*, and decreased the abundances of *Paraprevotella*, *Romboutsia*, *Clostridium sensu stricto, Fusobacterium,* and *Turicibacter* (*P* < 0.05 or *P* < 0.01).

### Spearman correlation analysis between genus species and serological indicators

**Fig. 9:**
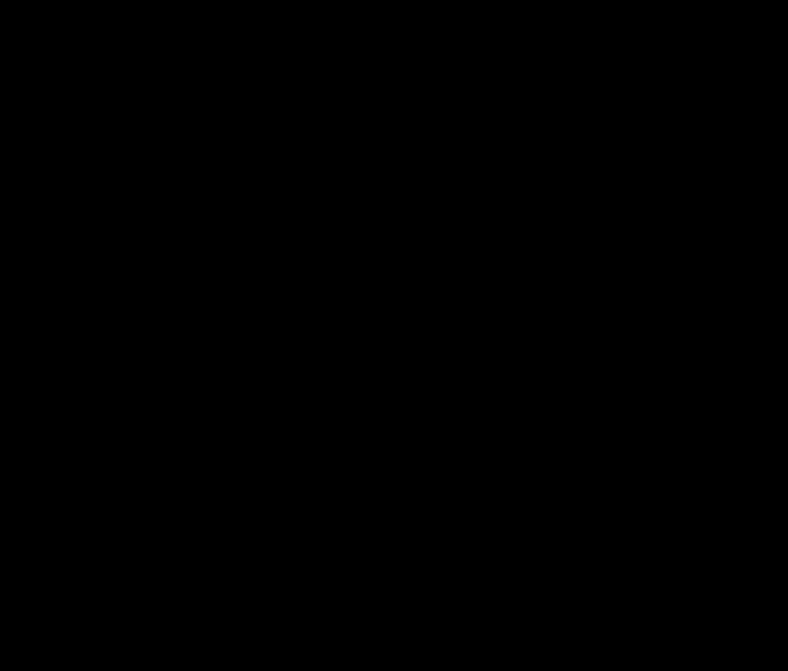
Spearman correlation analysis between genus species and serological indicators. The abundance of *Prevotella* was negatively correlated with serum ALT. The abundance of *Methanobrevibacter* was positively correlated with AST and TG. The abundance of *Clostridium sensu stricto* was positively correlated with ALT, TG, and LDL-CH. In addition, the abundances of *Romboutsia, Fusobacterium,* and *Turicibacter* were positively correlated with TG. X-axis, serological indicators; Y-axis, genus species. The depth of color visually shows the correlation between genus species and serological indicators. ^+^*P* < 0.05; *P < 0.01.

As shown in Fig. 9, the abundance of *Prevotella* was negatively correlated with serum ALT. The abundance of *Methanobrevibacter* was positively correlated with AST and TG. The abundance of *Clostridium sensu stricto* was positively correlated with ALT, TG and LDL-CH. In addition, the abundances of *Romboutsia Fusobacterium* and *Turicibacter* were positively correlated with TG. The results indicated that fucoidan may repair alcohol-induced liver dysfunction and lipid metabolism disorders by regulating gut flora.

## Discussion

The present study evaluated the effects of dietary fucoidan from the brown seaweed *Fucus vesiculosus* on liver injury and steatosis induced by long-term alcohol exposure in mice. The results showed that dietary fucoidan had a hepatoprotective effect in male C57BL/6J mice that were fed ethanol over a period of 8 weeks. Fucoidan treatment decreased serum ALT, CHOL, and hepatic TG levels. In addition, the morphology of hepatic cells was improved after fucoidan treatment.

The AMPKα1/SIRT1 pathway plays a key regulatory role in alcohol-induced oxidative stress response and lipid accumulation in hepatocytes (26). Our study showed that fucoidan could upregulate the AMPKα1/SIRT1 signaling pathway. Adenosine 5’-monophosphate (AMP)-activated protein kinase (AMPK) being a receptor for energy metabolism, is activated by many cellular stress responses such as limited nutrient intake, hypoxia, and oxidative stress. Phosphorylation of AMPK regulates its downstream molecules to maintain energy metabolism homeostasis (27). Previous studies have shown that AMPKα1 activates SIRT1 by increasing the levels of NAD^+^ and regulates energy metabolism, inflammation, and oxidative stress response *in vivo* by targeting SIRT1 (28, 29). SIRT1 is a widely expressed mammalian class III histone acetylation enzyme that requires NAD^+^ as a prosthetic group for acetylation of lysine (30). SIRT1 plays important roles in energy metabolism, oxidative stress balance, inflammation, DNA damage repair, and apoptosis, and also participates in ileal bile acid absorption to maintain bile acid homeostasis (31, 32).

Many rodent and human studies showed that impairment of hepatic SIRT1 signaling was closely associated with obesity and ALD (33, 34). Activated SIRT1 has a regulatory effect on hepatic lipogenesis, fatty acid oxidation, oxidative stress, and inflammatory response via regulation of the downstream molecules PGC-1α, PPARα, and HNF-1α (35, 36). Han et al. (37) reported that Korean red ginseng could reduce alcohol-induced oxidative stress and lipid accumulation in liver cells via the AMPK/SIRT1 pathway. Wang et al. (38) found that nicotinamide riboside attenuated ALD through the SIRT1/PGC-1α pathway.

SIRT1 regulates the expression of ChREBP protein, as demonstrated by the upregulation of ChREBP in specific SIRT1 knockout mice, which led to imbalance of lipid utilization and lipid excretion, accompanied by severe hepatic steatosis (39). ChREBP is a transcriptional regulator of glucose metabolism, mainly involved in the transcriptional activation of glycolysis and genes related to lipid-generation. In the absence of nutrients, SIRT1 inhibits transcriptional activation of the HNF-1-mediated C-reactive protein (CRP) promoter (40). Previous studies have shown that HNF-1α can bind to the promoter region of CRP and regulate its expression. Our data revealed that fucoidan could downregulate the levels of HNF-1α, IL-6, and IL-18. In the obesity model, SIRT1 induced transcriptional activation of PGC-1α and increased the expression levels of target antioxidant genes, alleviating the damage caused by oxidative stress (41). Our study showed that fucoidan upregulated PGC-1α expression and downregulated the expression of CYP2E1, Grp78, and 3-NT in the liver. We demonstrated that fucoidan treatment suppressed the expression of ChREBP and HNF-1α and upregulated PGC-1α by activating the AMPK-α1/SIRT1 pathway to exert its anti-inflammatory and antioxidant effects and suppress lipid accumulation, thereby alleviating chronic alcoholic liver injury.

Moreover, SIRT1 is a key nutrient sensor that is involved in systemic bile acid homeostasis and ileal bile acid absorption in mice. Kazgan et al. (42) demonstrated that systemic bile acid homeostasis was altered and HNF-1α–FXR signaling was impaired after deletion of SIRT1 in mice. Hartmann et al. (43) reported that chronic ethanol administration upregulated bacterial choloylglycine hydrolase, an enzyme which deconjugates bile acids in the intestine. Chronic alcohol feeding also decreased the secretion of FGF15 molecule in the small intestine and increased hepatic bile acid synthesis. Chronic alcohol administration elevated hepatic and serum bile acid levels, which synergized with alcohol to increase the severity of liver damage. In the present study, we found that ethanol feeding increased serum and hepatic TBA levels. However, treatment with fucoidan resulted in a decline in hepatic TBA levels. Moreover, ethanol-fed mice displayed higher fecal CA and MCA levels but lower DCA, TDCA, and GDCA levels as compared to control mice. Fucoidan reduced the levels of CA and MCA but increased TDCA levels. The bile acid profiles of the fucoidan intervention group were similar to that of the control group. These results suggest that fucoidan could ameliorate the disorder of bile acid metabolism caused by alcohol administration.

FGF15 is an ileum-derived hormone that can regulate inflammatory response, lipid metabolism, and bile acid synthesis (44). FGF15 can bind and stimulate hepatic fibroblast growth factor receptor 4 (FGFR4)/β-Klotho, subsequently inhibiting CYP7A1. It has been demonstrated that chronic alcohol administration in mice downregulated ileac FGF15 (45). Accordingly, we observed that the serum level of FGF15 was reduced in ethanol-fed mice as compared to control mice. Lower ileac FGF15 expression in ethanol-fed mice could potentially be mediated by increased reabsorption of bile acids (46). Our data showed that fucoidan significantly reversed the ethanol-induced reduction of ileac FXR and FGF15 levels and decreased CYP7A1 levels in hepatic tissues to relieve liver steatosis. Therefore, the protective effect of fucoidan is partly mediated by the CYP7A1 inhibition in the liver. It has been reported that upregulation of FGF15 was observed after mitoNEET deficiency in mice and it could protect against alcoholic steatohepatitis (47, 48), which is consistent with our results.

In addition, it has recently been reported that bile acid homeostasis is a function of microbial bile acid metabolism, and the regulatory effect of microbiota on bile acids plays a key role in the development and progression of ALD (49). It has been reported that the composition of gut microbiome was shifted to mostly members of the gram-positive Firmicutes by cholic acid feeding. Gut flora played a role in regulating the rate-limiting enzymes in bile acid synthesis in the liver (50). In this study, alcohol feeding decreased the abundances of *Prevotella*, *Lactobacillus*, and *Ruminococcus*, and increased the abundances of *Alloprevotella*, *Clostridium* XI, *Escherichia*, *Paraprevotella*, *Methanobrevibacter*, and *Treponema*. The genus distribution in fucoidan group was similar to that in the control group. Fucoidan reduced the abundance ratio of Firmicutes/Bacteroidetes, indicating that fucoidan could ameliorate ethanol-induced disruption of the gut microbiota. Fucoidan regulates the structure of gut flora by increasing the abundance of *Prevotella*, and decreased the abundances of *Paraprevotella*, *Romboutsia* and *Clostridium sensu stricto*. These results suggest that fucoidan-induced alteration of gut flora may not only be related to the maintenance of bile acid homeostasis but will also promote the enrichment of bacteria that produce short-chain fatty acids and inhibit other bacteria, including those that can produce endotoxins.

In conclusion, this study revealed that fucoidan inhibited alcohol-induced steatosis and disorders of bile acid metabolism via the AMPK/SIRT1 pathway and the gut microbiota–bile acid–liver axis. The data suggest that fucoidan may be effective in the prevention and treatment of ALD.

## Conflict of interest and funding

The authors declare no conflicts of interest.

## Supporting information

S1 Excel. ELISA data. The levels of triglyceride (TG), total cholesterol (CHOL), low-density lipoprotein cholesterol (LDL-CH), high-density lipoprotein cholesterol (HDL-CH) and total bile acid (TBA) in the serum and liver homogenates were quantified using ELISA kits.

S2 Excel. The fecal bile acid analysis performed by LC-MS.

S3 Excel. The abundances of bacteria at the phylum level. Alcohol-feeding increased the abundances of Proteobacteria and Euryarchaeota phylum.

S4 Excel. The abundances of bacteria at the genus level.
